# Recovery from Food Waste—Biscuit Doughs Enriched with Pomegranate Peel Powder as a Model of Fortified Aliment

**DOI:** 10.3390/biology11030416

**Published:** 2022-03-09

**Authors:** Domenico Nuzzo, Pasquale Picone, Jesus Lozano Sanchez, Isabel Borras-Linares, Alessandro Guiducci, Emanuela Muscolino, Daniela Giacomazza, Tiziana Sanfilippo, Rossella Guggino, Donatella Bulone, Clelia Dispenza, Pier Luigi San Biagio, Romano Lapasin

**Affiliations:** 1 Istituto per la Ricerca e l’Innovazione Biomedica, Consiglio Nazionale delle Ricerche, 90146 Palermo, Italy; pasquale.picone@irib.cnr.it; 2 Department of Food Science and Nutrition, University of Granada, 18071 Granada, Spain; jesusls@ugr.es; 3 Center of Research and Development of Functional Food, Health Science Technological Park, 18100 Granada, Spain; iborras@ugr.es; 4 IEMEST-Istituto Euro-Mediterraneo di Scienza e Tecnologia, 90139 Palermo, Italy; ale.guiducci@hotmail.com; 5 Dipartimento di Ingegneria, Università degli Studi di Palermo, 90128 Palermo, Italy; emanuela.muscolino@unipa.it; 6 Istituto di Biofisica, Consiglio Nazionale delle Ricerche, 90146 Palermo, Italy; donatella.bulone@cnr.it (D.B.); pierluigi.sanbiagio@cnr.it (P.L.S.B.); 7 Anestesia e Rianimazione, Presidio Ospedaliero “Salvatore Cimino”, 90141 Palermo, Italy; dott.tiziana.sanfilippo@gmail.com (T.S.); gugginorossella@libero.it (R.G.); 8 Ambulatorio di Nutrizione Clinica, ASP Palermo, 90141 Palermo, Italy; 9 Dipartimento di Ingegneria e Architettura, Università degli Studi di Trieste, 34127 Trieste, Italy; romano.lapasin@dia.units.it

**Keywords:** pomegranate peel, fortified food, food waste recovery

## Abstract

**Simple Summary:**

The rapid increase in consumer demand for foods rich in beneficial health effects pushes the manufacturing industries to identify molecules that can contribute to food quality in terms of nutritional values, stability, safety, and health benefits. Longevity is associated with healthy food intake, and consumers want to be aware of the nature of the molecules present in their food or, even better, of the molecules contributing to the enrichment of their food. Furthermore, the use of industrial food processes that tend to protect the environment are a further factor that contributes to the consumer’s interest.

**Abstract:**

The aim of the present work is the characterization of biscuit doughs enriched with pomegranate peel powder (PPP) at 3 (PPP3) and 5 (PPP5) wt% in the prospect of developing a fortified aliment as a support of the therapy of chronic inflammatory diseases of the intestinal tract. The total phenolic content of the powder was preliminarily evaluated. Then, the main compounds present in the PPP were identified by HPLC-ESI-TOF-MS analysis, being mainly hydrolysable tannins. The PPP was then treated at 180 °C for 20 min to mimic the baking treatment, and its water-soluble fraction (PPPwsf) was then added in the Caco-2 cell culture as a model of the intestinal epithelial barrier to verify its dose-dependent toxicity, ability in counteracting the oxidative stress, and anti-inflammatory action. Rheological experiments were performed to predict the macroscopic behavior of the PPP-added doughs during lamination and biscuit baking. SEM investigations gave their contribution to the microscopic comprehension of the dough structure. Finally, a consumer panel composed by thirty volunteers was enrolled to express its opinion on the sensory agreeableness of the biscuits prepared with two different concentrations of PPP compared with the reference dough. The discussion is focused on the biological effects of the main components found in the PPP.

## 1. Introduction

The excessive amount of food loss and waste strongly impacts the agriculture and climate change, being the major source of the community’s solid wastes [1]. In fact, the accumulation of wastes in landfills, in addition to requiring even larger spaces, causes the release of methane gas produced by microorganisms during the anaerobic digestion of the compostable by-products. On one hand, this process has in the past opened the route to the recycling of biogas; on the other hand, if not well managed, it is 84 times more responsible for global warming emissions than the carbon dioxide [1,2]. Wastes derived from fruits primarily contain cellulosic residues and, for this reason, they are the favorite substrate in the anaerobic process for biogas production [1]. Thus, reducing and recycling food by-products is important for the environment, the agriculture, and the recovery of the spaces destined for landfills.

Over the last decades, customers have been requesting healthy food containing active ingredients to delay aging and prevent diseases. Thus, while the scientific research is illustrating their beneficial properties [3,4,5], introducing the concept of “food as a medicine” [6], enriched and fortified foods have made their appearance on the market shelves. It is worth noting that the term “enriched food” indicates alimentary products in which elements that are originally present in the raw materials but are lost or deteriorated during processing have been added, while the term “fortified food” identifies foods in which bioactive molecules, such as many phytocompounds, not present in the original ingredients, have been introduced. Most of the success of phytocompounds comes from their antioxidant properties. These molecules have the ability to neutralize or quench the chemical reactions, leading to free radical production. The intake of foods containing these molecules helps to reduce inflammatory processes, improving lipid profiles and reducing the risk of chronic disease. Furthermore, phytocompounds present in many agri-food by-products still possess relevant functional biological activities [7,8] that contribute in the treatment of neurodegeneration [9], metabolic syndrome [10], cardiovascular diseases [11], cancer [12], and against virus-related infections [13]. Antimicrobial properties have also been found in food waste from citrus waste [14,15].

Longevity is associated with healthy food intake, and recent research in vitro and in vivo have demonstrated that the length of telomers, which directly affect the lifespan, is deeply modified by antioxidant treatment [16,17].

Thus, today the consumers want to be aware of the nature of the molecules present in their food or, better yet, to know the bioactive molecules that make food a healthy food.

Pomegranate (*Punica granatum* L.) is a small plant belonging to the Punicaceae family, originally cultivated in India and Iran, from where cultivation later extended to the entire Mediterranean basin. The edible part of the fruit corresponds to about 40% of the total weight, while the seeds and peel account for 10 and 50%, respectively [18]. The plant has been appreciated since ancient times for its effects on human health. It has also been mentioned in the Bible as a sacred fruit capable of bestowing luck, health, and fertility [19].

Pomegranate exhibits potent antioxidant, antibacterial, antifungal, antimicrobial, and antihypertensive effects [20,21,22,23,24]. It has also been reported that pomegranates contribute to the lowering of the blood lipid levels [25] and have significant antitumor activity, particularly against prostatic cancer [26].

The aim of the present work is to study the biological effects of the addition of the pomegranate peel powder (PPP) in the dough prepared for biscuits. Recent studies have illustrated the potential of fortified biscuits. The partial replacement of fats of animal origin or margarine, used in the original recipes of Italian Cantuccini biscuits with extra virgin olive oil, has allowed for the greater stability of the product during storage [5]. The addition of the byproduct obtained from instant coffee to the biscuit dough seems to be recommended for obese or diabetic patients as a low-fat food [3]. *Opuntia ficus-indica* L. peel flour was a successful partial substitute for sugar and gluten in the preparation of biscuits, increasing their content of bioactive molecules, fibers, and textural properties [4]. A comprehensive discussion regarding the incorporation of nutraceutical ingredients in baked food can be found in Hayta and Polat, 2014 [6].

In the present study, the total phenolic content of the PPP obtained from a biological farm was initially evaluated. Then, the single compounds present in the alcoholic fraction of PPP, treated at 180 °C for 20 min to simulate the baking treatment, were identified by HPLC-ESI-TOF-MS analysis as belonging prevalently to the hydrolysable tannin chemical class. The PPP water-soluble fraction (PPPwsf) was then added in the Caco-2 cell culture as a model of the intestinal epithelial barrier to verify its dose-dependent toxicity, ability in counteracting the oxidative stress, and anti-inflammatory action. Rheological and SEM experiments gave their contribution to predict the macroscopic behavior of the PPP-added doughs during lamination and baking and to the microscopic comprehension of the dough structure, respectively. Finally, a consumer panel composed of thirty volunteers was enrolled to express its opinion on the sensory agreeableness on the biscuits prepared with two different concentrations of PPP compared with the reference dough.

## 2. Materials and Methods

### 2.1. Materials

The pomegranate peel powder was purchased from Packtin, s.r.l. (Reggio Emilia, IT). The plants from which the powder was obtained belong to the Wonderful and Ako cultivars, with an average age of 10 years, grown in organic farming. Fertilizers with very similar values of potassium, phosphorus, and nitrogen (20 g per plant every 2 months) were used. The soil was drained to avoid stagnation. Once produced, the PPP was stored up to 18 months in a cool and dry place.

Nitric acid was purchased from Merck (Darmstadt, Germany). Methanol, acetonitrile, 2-propanol were purchased from Thermo Fisher Scientific (Waltham, MA, USA). Folin–Ciocalteu solution, sodium carbonate, gallic acid, methanol (HPLC grade), acetonitrile (LC-MS grade), phosphoric acid 85%, Luperox^®^ TBH70X tert-butyl hydroperoxide solution (TBH) and 20,70-dichlorodihydrofluorescein diacetate (DCFH-DA) were purchased from Sigma–Aldrich (Milan, Italy). The chemicals and solvents were analytical grade reagents or LC-MS grade. CellTiter 96^®^ Aqueous One Solution Assay (MTS) was purchased from Promega (Milan, Italy). The water used in all experiments was obtained from Millipore Milli-Q (Millipore, Bedford, MA, USA). All sample solutions were stored at −20 °C until analysis and were used within three days of preparation.

### 2.2. Biological Evaluation

#### 2.2.1. Total Phenolic Content

A conventional solid–liquid extraction was used to evaluate the biophenols contained in PPP before and after its thermal treatment at 180 °C for 20 min. A total of 3 g of the sample was added with 30 mL of 80:20 ethanol:water extraction solvent, kept for 30 min and decanted. The supernatant phase was magnetically stirred at room temperature for 1 h. Then, the suspensions were centrifuged at a relative centrifugal force (RCF) of 12,499 for 15 min at 4 °C in a Sorvall ST 16 R centrifuge (Thermo Scientific, Leicestershire, UK) and the supernatants dried at 35 °C in the Savan SC250EXP Speed-Vac (Thermo Scientific, Leicestershire, UK). The total phenolic amount present was measured by the Folin–Ciocalteu assay in agreement with Hrncirik and Frische [27]. The flavonoid content was determined as described in [28]. The extraction procedure was carried out in triplicate.

#### 2.2.2. HPLC-ESI-TOF-MS Analysis

PPP was treated as described in [28]. The gradient elution for resolving the PPP compounds was as follows: initial conditions 5% *v*/*v* B; 5 min, 15% *v*/*v* B; 10 min, 20% *v*/*v* B; 14 min, 22% *v*/*v* B; 17 min, 24% *v*/*v* B; 20 min, 26% *v*/*v* B; 23 min, 29% *v*/*v* B; 25 min, 55% *v*/*v* B; 32 min, 95% *v*/*v* B; 35 min, 5% *v*/*v* B; 40 min, 5% *v*/*v* B. The flow rate of mobile phase was 0.5 mL/min. The temperature of the column was maintained at 25 °C and the injection volume was 10 μL.

Detection was performed as in [28].

Data acquisition and processing for chemical characterization of PPP were controlled with HyStar 3.2 and Data Analysis 4.0 software, respectively (Bruker Daltonics, Bremen, Germany). The compound identification was done by using the molecular formula provided through parallel verification of software, literature information [29], and a database such as SciFinder, Scopus, or SciDirect.

#### 2.2.3. Extraction of Pomegranate Peel Powder Water-Soluble Fraction (PPPwsf)

PPPwsf was obtained from PPP after thermal treatment at 180 °C for 20 min by dissolving 10 mg of PPP in 1 mL of PBS buffer (pH = 7.4; 137 mM NaCl, 2.7 mM KCl, 8 mM Na_3_PO_4_). The suspension was sonicated twice in an ultrasonic bath at 59 kHz and 198 W for 30 s and magnetically stirred for two hours at room temperature. The insoluble fraction was removed after centrifugation at 2500 rpm for 15 min at 4 °C and resulted in about 55 wt% of the total weight of the sample and mainly consisted of fibers, proteins, and ash [30]. The collected supernatant was filtered (0.45 μm Sartorius filter), aliquoted (1 mL/vial), and stored at −20 °C for further analyses.

#### 2.2.4. Cell Cultures and Treatment

Caco-2 cells were cultured in T25 tissue culture flasks in DMEM (Corning) were used, supplemented with 10% *v*/*v* fetal bovine serum (FBS), 100 U/mL penicillin and 100 U/mL streptomycin (Sigma) and 2 mM l-glutamine in humidified atmosphere of 95% *v*/*v* air and 5% *v*/*v* CO_2_ at 37 C. Caco-2 cells were allowed to grow to confluence and were immediately used, undifferentiated, for biological experiments.

#### 2.2.5. Cell Viability and Morphology

Cells cultures and viability were performed as reported in [28]. Reported viability data were obtained as the percentage with respect to the control value. The PPPwsf toxicity evaluation was done by plating the cells in a 96-well and treating them with 100 μg/mL, 200 μg/mL, 400 μg/mL, 500 μg/mL of PPPwsf (hereafter identified as PPPwsf100, PPPwsf200, PPPwsf400, and PPPwsf500) for 24 h. In the control set, an equal volume of PBS was added. For the morphological analysis, cells were grown at a density of 5 × 10^3^ cell/well on a 96-well plate in a final volume of 100 μL/well and were washed twice with PBS. The cellular images were obtained using a Zeiss Axio Scope 2 microscope (Carl Zeiss, Oberkochen, Germany).

#### 2.2.6. PPPwsf Effect against ROS

The oxidative stress was generated by an addition of 2 mM of tert-butyl hydroperoxide (TBH, Luperox^®^ TBH70X, Merck). The stimulation was conducted for 24 h, alone and in mixture with PPPwsf. The control groups (Control) received an equal volume of PBS. Then, each sample was added to 100 µM of DCFH-DA (Abcam, Italy) and placed in the dark for 10 min at 37 °C. After washing twice with PBS, cells were analyzed using a fluorimeter Microplate Reader GloMax (Promega Corporation, Italy) at the excitation and emission wavelengths of 475 nm and 530 nm, respectively. Results were expressed as a fluorescence intensity.

#### 2.2.7. Genotoxicity Assay

To determinate the genotoxicity of PPPwsf, the cells were incubated for 24 h with the extracts at different concentrations. At the end of the experiment, the medium was removed, and cells were washed twice with PBS and subsequently stained with acridine orange/PBS solution (Merck, Italy) at 100 μg/mL for 10 min in the dark at room temperature. After three washes in PBS solution, the cells were analyzed using a fluorimeter Microplate Reader GloMax (Promega Corporation, Italy) at the excitation wavelength of 475 nm and emission wavelengths 526 nm and 650 nm for single-stranded DNA (ssDNA) and double-stranded DNA (dsDNA), respectively. Results were expressed as the dsDNA/ssDNA fluorescence intensity ratio using the control fluorescence intensity as reference. Cell fluorescence was also visualized using the fluorescence microscope Zeiss Axio Scope 2 microscope (Carl Zeiss, Germany). TBH at a concentration of 2 mM was used as a positive control.

#### 2.2.8. Immunological Tests

The peripheral blood mononuclear cells (PBMCs) were isolated from 5 mL of venous blood collected early in the morning from five healthy donors with an age range of 40–45 years old (Ethics Committee number 8/2021, 15 September 2021). PBMCs were isolated from heparinized blood by Lymphosep (Biowest) and cultured at a concentration of 3 × 10^5^/mL cells in a 24-well, flat-bottom plate in complete RPMI 1640 medium supplemented with 10% FBS, 2 mM glutamine, and 100 U/mL penicillin/streptomycin at 37 °C. PBMCs were not treated (control) or were 4-h treated with PPPwsf at a concentration of 200 µg/mL with or without lipopolysaccharide (LPS) at a concentration of 1 µg/mL. At the end of the treatment, the cells were collected for RNA extraction and the supernatant was submitted to IL1β Enzyme-Linked Immunosorbent Sandwich Assay–ELISA (Sigma–Aldrich) according to manufacturer instruction.

#### 2.2.9. Quantitative Real-Time qPCR

Total RNA was extracted using High Pure RNA Isolation Kit (Roche). One µg of RNA was used to synthesize the cDNA using iScript cDNA Synthesis Kit (Bio-Rad). Synthesized cDNAs were amplified using SsoAdvanced Universal SYBER Green Supermix (Bio-Rad) by StepOne Real-Time instrument (Applied Biosystem). Gene expression validation was performed using home-made sequence primers for human TNFα, Interleukin 1 beta (IL-1β), Interleukin 8 (IL-8), and β-actin (Table 1). Gene expression was normalized to β-actin. The relative expression levels of RNA were calculated by StepOne™ software by using the comparative CT (ΔΔCT) quantitation method.

#### 2.2.10. Statistical Analysis

All values reported were obtained as the mean of at least three independent experiments ± standard errors (SE). Results were compared using one-way analysis of variance with pairwise comparisons among treatments made using *t*-test. The analyses were per- formed using the SigmaPlot 11.0 statistical program (Systat Softwar, San Jose, CA, USA). Results were considered statistically significant at *p* < 0.05.

### 2.3. Biscuit Dough Preparation and Characterization

#### 2.3.1. Biscuits Composition and Preparation

The biscuits were produced by a local company (“Natisani s.r.l.”, Palermo, Italy) producing food for celiac subjects. Dough ingredients in descending amount were rice flour, potato starch and modified potato starch (51%), sucrose (18.4%), vegetable margarine (10.5%), egg (9%), water (7.5%), glucose powder (2.3), locust bean gum and guar gum (1%), and baking powder (0.3%). The chemical composition, as indicated by the producer company, was carbohydrates 64 wt%, fats 12 wt%, proteins 4.5 wt%, fibers 2 wt%, and minerals 0.15 wt%.

The biscuits were prepared as follows: as a first step, rice flour, starches, sucrose, glucose powder, water, locust bean gum, and guar gum were mixed in the planetary. Then, margarine and baking powder were added. Finally, eggs were added to the dough. Mixing was continued until a homogeneous mixture was obtained. The dough was kept for 1 h at 4 °C before being used.

PPP was added at a concentration of 3 wt% and 5 wt% instead of the rice flour ingredient and indicated as PPP3 and PPP5, respectively. The blank reference dough indicated as BD. For the biscuit preparation, the doughs were kept in the oven at a temperature of 180 °C for 20 min. After baking, the biscuit dimensions were 0.7 cm thickness and 4.5 cm diameter.

#### 2.3.2. Rheological Measurements

Dynamic tests were carried out on the fresh raw doughs using an AR-G2 (TA Instruments, New Castels, DE, USA) rheometer equipped with an acrylic plate geometry (diameter: 40 mm, gap height ranging from 2500 to 4000 μm) at a temperature of 25 ± 0.1 °C, controlled with a built-in Peltier system. The strain sweep measurements were performed at constant frequency (1 Hz) in the strain range 5 × 10^−6^ ÷ 1. The frequency sweeps were performed in the LVE region at constant strain (8 × 10^−5^) in the frequency range 0.02 ÷ 100 Hz.

#### 2.3.3. SEM Morphological Analysis

Fresh raw dough microstructures were investigated using a Field Emission Scanning Electron Microscope (SEM) Phenom ProX desktop at an accelerating voltage of 15 kV. The samples were frozen at −20 °C, freeze-dried, mounted on aluminum stubs, and were gold coated by JFC-1300 gold coater (JEOL) for 120 s at 30 mA before scanning.

#### 2.3.4. Panel Test

Thirty volunteer subjects, trained in the use of the sensory profiles method, were enrolled for the sensory characterization of the biscuits obtained with the blank dough and the doughs containing PPP3 and PPP5 [19]. Panel members (60% women and 40% men) were aged between 20 and 65; 2 were smokers; 8 were researchers, 12 laboratory technicians, 6 employers, and 4 PhD students. The blind panel expressed its opinion on the tested products through the evaluation of nine different descriptors (color, smell, sweetness, sapidity, compactness, friability, crunchiness, adhesion to the palate, and persistence of flavor), weighing the appearance, the flavor and the chewiness of the products. The evaluation was done by giving a score from 1 (very low intensity) to 5 (very high intensity) for each descriptor.

## 3. Results

### 3.1. Total Phenolic Content

The total phenolic content was preliminarily evaluated in the PPP. Results (Table 2) indicated that the PPP is highly rich in antioxidant molecules. It should be noted that the extra virgin olive oils, particularly appreciated for their phenolic content, contain between 200 and 800 mg/kg of these molecules, being this value is strongly affected by several parameters, such as the geographical area of the cultivated land, olive cultivar, fruit ripening and harvesting, agronomic procedure, and technological operation for oil production [31].

### 3.2. HPLC-ESI-TOF-MS Analysis

**Figure 1:**
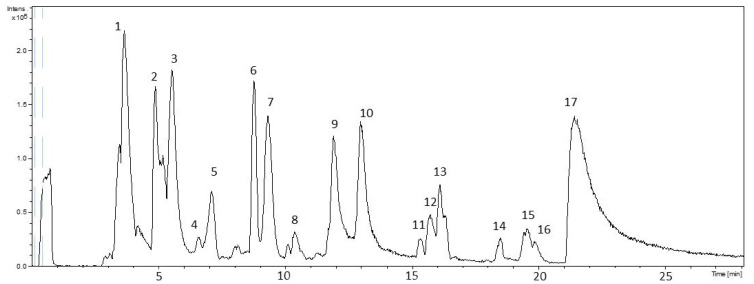
HPLC-ESI-TOF-MS chromatogram of the PPP.

To the purpose of identifying the molecules present in the PPP, an HPLC-ESI-TOF-MS measurement was performed, allowing the detection of a total of 17 phytochemical compounds (Figure 1 and Table 3). Twelve compounds, previously reported in pomegranate by-products [30], were classified according to their chemical nature in the following families: sugars, organic acids, phenolic acids, and hydrolysable tannins.

### 3.3. Biological Effects of the PPPwsf

The in vitro evaluation of the effects of the bioactive compounds present in the PPP was performed by collecting the water-soluble fraction obtained from the whole PPP after its thermal treatment at 180 °C for 20 min.

Previous work has indicated that there is no significant difference between water- and alcoholic-extracts of PPP [32,33] used for the HPLC-ESI-TOF-MS experiments. These results suggested that the chemical molecules identified in the HPLC-ESI-TOF-MS analysis continued to be present in the PPPwsf used for biological experiments.

**Figure 2:**
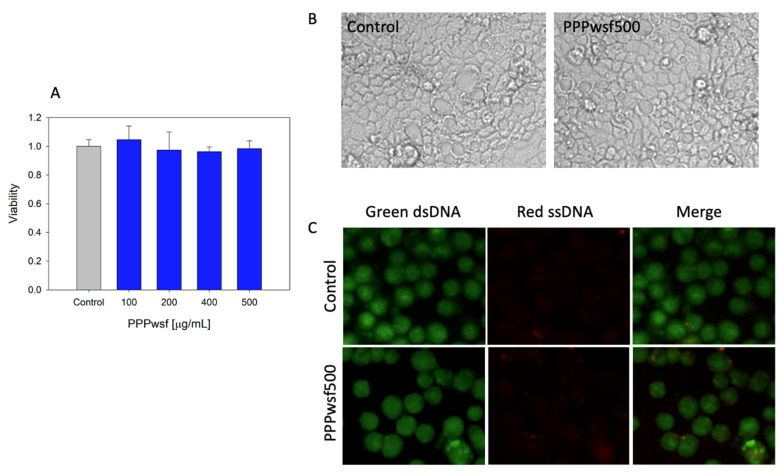
Cytotoxicity test of the PPPwsf solutions: (**A**) Cell viability 24 h after the addition of PPPwsf at the concentration of 100 μg/mL, 200 μg/mL, 400 μg/mL, 500 μg/mL in Caco-2 cell culture. Data were obtained as a percentage with respect to the control sample. (**B**) Visual inspections of the morphology of Caco-2 cells 24 h after the addition of PPPwsf at the concentration of 500 μg/mL. The morphology of the control is also reported. Magnification 40×. (**C**) Microscopic fluorescence visualization of the morphology of Caco-2 cells 24 h after the addition of PPPwsf at a concentration of 500 μg/mL after staining with orange acridine. Data concerning the control were also reported. Magnification 20×.

To study the effect of PPPwsf, undifferentiated Caco-2 cells were used. This choice was justified, considering that differentiated Caco-2 cells present a high cell line variability that may significantly affect their response to the same molecules [34]. As a first step, the toxicity analysis was done to detect the concentration range that is nontoxic for the cells. In Figure 2A, the results of the toxicity tests obtained after the addition of the Caco-2 cell model of four PPPwsf solutions containing pomegranate peel powder at concentrations of 100 μg/mL, 200 μg/mL, 400 μg/mL, and 500 μg/mL are reported. The solutions were kept 24 h in direct contact with the cells. Results indicated that in the concentration range explored neither the viability of the cells nor their morphology; monolayer integrity and organization aptitude were affected by the addition of PPPwsf, even at the higher concentration used. (Figure 2B). In Figure 2C the microscopic fluorescence images concerning the signal induced by the acridine orange fluorescent probe are presented. This is a cell permeating nucleic acid binding dye that emits green fluorescence when bound to double-stranded DNA and red fluorescence when linked to single-stranded DNA and is thus able to discriminate between intact (green nuclei) and damaged DNA, that is between alive and dead cells [35]. As evidenced by the largely dominant green fluorescence, after the addition of PPPwsf at the highest concentration (500 μg/mL), a negligible DNA damage was detectable in the samples.

**Figure 3:**
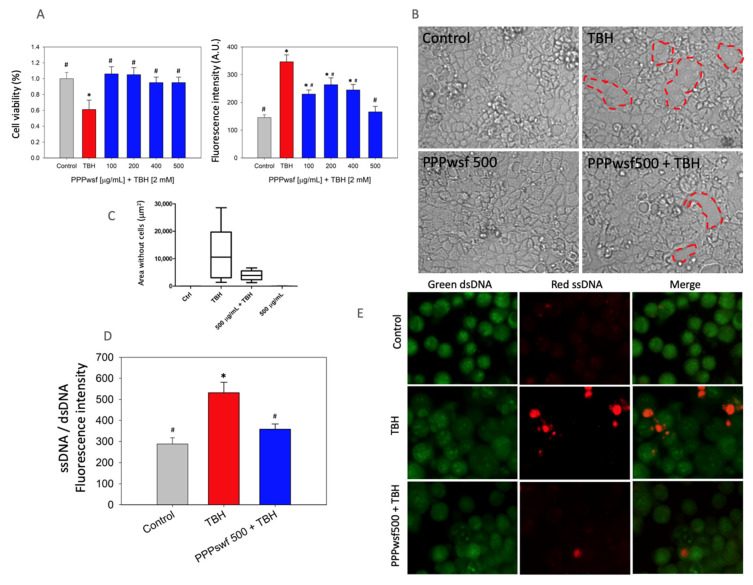
PPPwsf co-treatment in Caco-2 cells: (**A**, left) Cell viability of Caco-2 cells and (**A**, right) fluorescence intensity values due to the ROS presence in Caco-2 cells, 4 h after the addition of PPPwsf at the concentration of 100 μg/mL, 200 μg/mL, 400 μg/mL, and 500 μg/mL together with TBH 2 mM. Cell viability data are reported as percentage with reference to the control value. * Control vs. other treatments (*p* ≤ 0.05); # TBH samples vs. control and other treatments (*p* ≤ 0.05). (**B**) Microscopic inspection of the Caco-2 cell layer treated with TBH alone (positive control) and after the complete co-treatment with PPPwsf500 and TBH 2 mM. The dashed red lines circumscribe the area without cells. Control and PPPwsf500-treated cells are also reported. (**C**) Plot of the values of the areas of the Caco-2 layer without cells. (**D**) Fluorescence intensity of the ratio between red and green fluorescence intensities in Caco-2 cells 4 h after co-treatment with PPPwsf500 and TBH 2 mM and staining with orange acridine. * Control vs. other treatments (*p* ≤ 0.05); # TBH samples vs. control and other treatments (*p* ≤ 0.05). (**E**) Microscopic fluorescence signals after orange acridine staining of the Caco-2 cells 4 h after co-treatment with PPPwsf500 and TBH 2 mM. Data concerning the control and TBH alone were also reported. Magnification 40×.

To test the scavenging ability of the PPPwsf solution, TBH was added to the cell culture together with PPPwsf at various concentrations, namely 100 μg/mL, 200 μg/mL, 400 μg/mL, and 500 μg/mL for 4 h, which is the time at which, at the intestinal level, the maximum biophenolic absorption, corresponding only to a fraction of the total intake, occurs [36]. Results indicated that the co-treatment was effective against ROS-induced cellular damage. In particular, it can be observed that: (i) no decrease in the cell viability was observed (Figure 3A, left panel); (ii) a remarkable decrease in the fluorescence associated with the ROS presence could be recorded in the cell culture, particularly at the highest PPPwsf concentration used (Figure 3A, right panel); (iii) although still present, breaks in the integrity of the epithelial layer were noticeably reduced when TBH was added to the cells together with PPPwsf (Figure 3B,C) when compared with the effect of TBH alone; (iv) the addition of acridine orange fluorescence allowed the authors to conclude that only a slight DNA damage was detectable, as evidenced by the value of the ratio between the fluorescence induced by the bond with the single and double stranded DNAs and the microscopic fluorescence investigation of the cells (Figure 3D,E).

**Figure 4:**
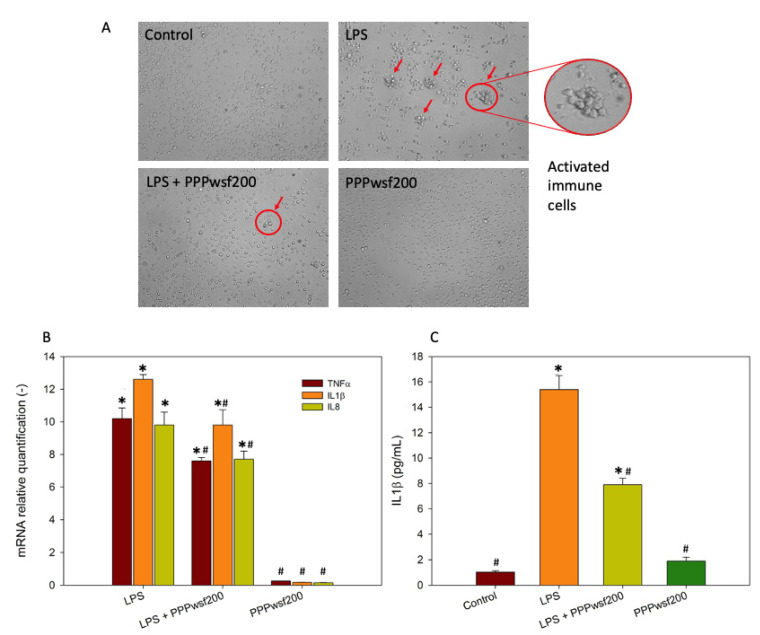
PPPwsf co-treatment in PBMCs: (**A**) Morphological analyses of cells activation by clustering formations after 4 h treatment with LPS 1 μg/mL, LPS 1 μg/mL + PPPwsf 200 µg/mL, and PPPwsf 200 µg/mL. Red circles indicate the clusters of activated immune cells. Magnification 20×. (**B**) Immunological response evaluated by mRNA quantification of TNFα, IL1β, and IL8. (**C**) Level of interleukin IL1β measured by ELISA assay. * Control vs. other treatments (*p* ≤ 0.05); # TBH samples vs. control and other treatments (*p* ≤ 0.05).

To verify the effect of the PPPwsf on inflammation, the 200 μg/mL concentration was chosen due its better response in counteracting ROS formation (Figure 3A, left). To this aim, the activation of the immune cells (Figure 4A) and the expression of some of the most highly expressed genes associated with inflammation, such as TNFα, IL1β and IL8, were evaluated (Figure 4B). During the 4 h co-treatment with PPPwsf200, significant changes in the levels of the expression of the proinflammatory genes were detected. In particular, our results revealed an up-regulation of the mRNA of the cytokines investigated. In addition, the concentration of IL1β protein (Figure 4C), the most critical cytokine of inflammatory processes [37], was significatively decreased when the cells were co-treated with PPPwsf, with respect to the LPS alone treatment. These data suggested that the mediators of inflammation are down-regulated in the presence of PPPwsf.

### 3.4. Biscuit Dough Preparation and Characterization

**Figure 5:**
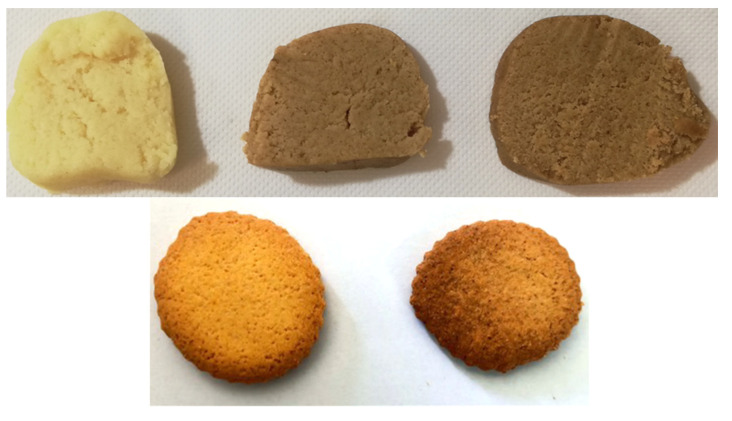
(**Upper** panel) Appearance of the raw doughs for biscuit preparation. (**Left**) BD, (**middle**) PPP3, (**right**) PPP5 doughs before baking at 180 °C for 20 min. (**Lower** panel) The BD (**left**) and PPP3 (**right**) doughs after baking.

In Figure 5 (upper panel), the appearance of the dough containing 3 (PPP3) and 5 (PPP5) wt% of PPP before heat treatment is reported and compared with the one of the base doughs (BD). The final products, after baking, are also represented (lower panel).

In Table 4, the concentration of the phenolic compounds found in the dough treated at 180 °C for 20 min and containing the 5 wt% of PPP is described. As evidenced, a significant amount of the bioactive molecules was present in the dough, notwithstanding the thermal treatment.

During the preparation of biscuits, the control of the viscoelastic parameters of the raw dough is of primary importance. On the one hand, a high elasticity can make difficult the dough lamination; on the other hand, a low viscosity can favor the spreading of the doughs during baking. Thus, rheological experiments performed under oscillatory shear conditions at low strain (mechanical spectra) as well as over an extended stress range (stress sweeps) can be useful to characterize the structural and textural properties of the dough.

**Figure 6:**
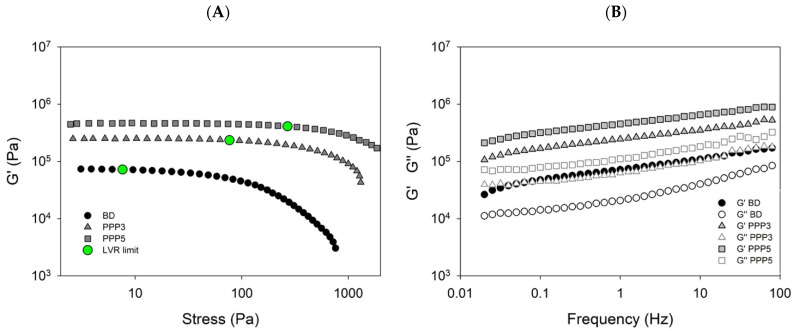
Rheological behavior of the raw doughs: (**A**) G’ vs. applied stress for the reference dough (BD) and the doughs containing the pomegranate peel powder at a concentration of 3 (PPP3) and 5 (PPP5) wt%. The full green dots show the stress values limiting the linear viscoelastic region. (**B**) Mechanical spectra illustrating the profiles of the elastic, G’ (full symbols), and viscous, G” (empty symbols), moduli as a function of the frequency for P3 (triangles) and P5 (squares) samples compared to the blank dough (circles).

In Figure 6, the rheological behavior of the reference dough and of the doughs added with 3 wt% and 5 wt% PPP are displayed as a function of the increasing stress (Figure 6A). For each sample, the limit of the linear viscoelastic region has been set corresponding to a value of G’ lower than 5% of its plateau value. It can be observed that the blank dough has a critical stress threshold (7.6 Pa) lower than those containing the pomegranate powder at both the concentrations used. Furthermore, for the samples in which the pomegranate peel powder is present, the stress values above which the viscoelastic response became nonlinear is at least one order of magnitude higher that that identified for the blank dough, ranging from 76.7 (PPP3) to 269 (PPP5) Pa. These results indicated that the addition of increasing amounts of powder caused a progressive mechanical reinforcement of the doughs. The profile of the mechanical spectra appeared slightly dependent on the frequency, with an appearance typical of solid-like materials. This behavior increased at increasing PPP content (Figure 6B). However, the dough containing the pomegranate peel powder remained a suitable material for lamination and baking [38].

**Figure 7:**
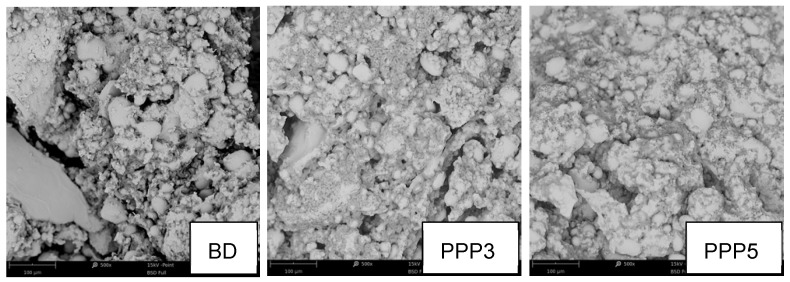
SEM images of the BD, P3, and P5 samples morphology. Magnification 500×.

The results illustrated by rheological measurements were confirmed by the SEM experiments performed on the BD, PPP3, and PPP5 raw doughs (Figure 7). The PPP5 sample presented the higher compactness degree; on the contrary, the BD sample showed a heterogeneous and coarse structure due to the existence of differently sized granules. Large fissures were also visible, interrupting the matrix. The PPP3 sample had an intermediate aspect between BD and PPP5: there were some small fissures interrupting the matrix continuum, but the appearance approached the homogeneity of the PPP5 sample.

**Figure 8:**
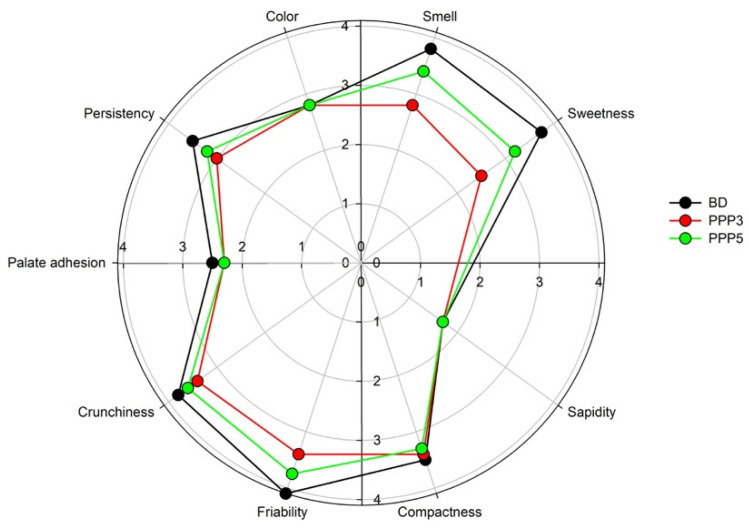
Blind panel results testing the sensory pleasantness of the biscuits prepared with the reference dough (BD) and the biscuits prepared with pomegranate peel powder at a concentration of 3 (PP3) and 5 (PPP5) wt%. The mean SD was ≤0.3.

A blind test was performed to test the appearance, taste, and chewiness of the PPP-containing biscuits in comparison to the PPP-free biscuits, representing the reference commercial product. Results (Figure 8) indicated a slight general preference of the panel for the sample without addition of PPP for each descriptor. In particular, the panelists did not perceive any difference among the samples for what concerned color, sapidity, palate adhesion, and compactness. Conversely, significant differences were found in the crunchiness, persistency, friability smell, and sweetness descriptors. The lowest rating was recorded for the biscuits containing the highest pomegranate peel powder concentration, probably due the excessive presence of polyphenols, which overall reduced the sensory pleasantness of the biscuit, and, for this reason, was excluded from the industrial production. The PPP3 product obtained a more than satisfactory approval from the panel members.

## 4. Discussion

The HPLC-ESI-TOF-MS phenolic profile illustrated in the present work was in perfect agreement with previous literature data [39]. Molecules belonging to the hydrolysable tannin group (such as different chemical forms of the punicalagin) and some punicalagin derivatives (such as chemical species of ellagitannins) were found. These large molecules are not absorbed in vivo but, after reaching the colon, are metabolized by the human microflora in urolithins. Corilagin, chlorogenic acid and gallic acid were also present.

After intake, the fate of the polyphenolic molecules is a complex mechanism. Their absorption at a gastric level is a rare event and pertains only to few molecular species such as hydroxycinnamic acids and anthocyanins [40].

Only in the small intestine can they be absorbed after the hydrolysis of the glycoside group, and two mechanisms have been proposed for this process. In the first one, the molecule is de-glycosylated inside the enterocyte after active absorption of the Na^+^ dependent glucose transporter. In the second proposed mechanism, the enzymatic hydrolysis takes place in the intestinal lumen from which the de-glycosylated product would passively enter the cells. Whatever the mechanism, only very few polyphenolic molecules are absorbed in the small intestine, while the rest is transferred to the colon, where it can reach a high concentration (up to millimolar) to be metabolized by the gut microbiota, which therefore plays a fundamental role in their absorption. The absorbed polyphenolic molecules are transported by the bloodstream to the liver, from which, through the bile, they return to the large intestine [41,42]. Interestingly, on the one hand, intestinal microbiota are able to modify and alter the biophenol composition; on the other hand, biophenols can in turn modify the gut microbiota and their balance [43].

The antioxidant ability of the PPPwsf was previously studied by Sorrenti and coworkers [44], testing its ability in reducing the stable DPPH (2,2-diphenyl-1-picrylhydrazil) radical. The inhibition activity was up to 75% for a 0.028 mg/mL PPPswf concentration. Moreover, a scavenging ability of 95% was observed at the same concentration with respect to the superoxide anion formation. These results were obtained by using the whole water-soluble extract of PPP. Analyzing pomegranate juices, Gil et al. observed that the antioxidant activity was higher in juices containing the whole fruit with respect to the ones obtained by the arils only [45]. Furthermore, the antioxidant activity of each isolated phenolic component and of each individual phenolic family measured with the DPPH method were higher for punicalagin and punicalagins, respectively. These data suggests that the results obtained in the present work can be ascribed to the same molecules identified by Gil et al.

Previous studies performed on muffin doughs containing PPP at the concentrations of 5 wt%, 10 wt%, and 15 wt% indicated that the total polyphenol content increased three-, five-, and seven-fold, respectively, compared with the dough without PPP. Furthermore, their antioxidant activity was increased 10-, 21-, and 27-fold, respectively [46]. Tomato juice and orange juice added with 2 wt% PPP showed an increase in the antioxidant power 25- and 35-times greater than the controls, respectively. However, it is important to note that, although in all the experiments in which PPP has been added a significant increase in the polyphenol content and of the antioxidant power are observed, the values can change because of the variability in pomegranate plants and their geographical area of cultivation. It has been observed that pomegranate arils grown in Mediterranean regions possess a higher concentration of antioxidant molecules compared to the plants cultivated in desert zones [47]. On the contrary, peels of the fruits grown in desert areas show higher levels of antioxidant molecules [48]. The phenolic profiles of pomegranates are strongly dependent on the part of the fruit analyzed. Negligible concentrations of punicalagin were observed in the arils of 12 different cultivars, while peels revealed very high levels of this molecule [39]. Furthermore, the cultivar has also a great importance in determining the phenolic profiles [48]. The agronomic procedures for plant cultivation, and the technological processes applied to obtain the powders, also play a key role in the final characteristic of the product [46].

The integrity of the intestinal epithelium, formed by a single layer of cells, is of primary importance to prevent and avoid the entrance of toxic and noxious agent into the mucosa and bloodstream. Recent research has demonstrated that the ellagic acid is particularly effective in strengthening the intestinal epithelium by reducing the expression of the claudin-4, -7, and -15 pore-forming proteins in Caco-2 cells [49], thus contributing to the epithelium integrity. Furthermore, in Caco2 cells, urolithin A reinforced the barrier function of the colonic epithelium by upregulation of the expression of proteins involved in the tight junctions through activation of the Nrf2-dependent pathways. An attenuation of colitis pathological status was also evidenced in preclinical models due to the recovery of barrier dysfunction [50]. This molecule was also effective in other parts of the human body; in fact, urolithin was able to reduce the ROS, inhibited the production of proinflammatory cytokines and increased the anti-inflammatory IL-10 cytokine in LPS-stimulated microglial cells [51].

Recent research has reported that punicalagin and ellagic acid inhibited in vitro the growth rate of HepG2 cells (hepatoma cells) in a dose- and time-dependent manner. No effect was recorded against normal liver cells [52]. The results could be obtained by retaining the cancer cells at the G1 phase and blocking their division in the G0 phase. A further effect of the punicalagin consisted in the inhibition of the DNA synthesis of the cancer cells during the S phase [52]. The antiproliferative effect of the pomegranate phenolic extract was also observed against human oral cancer. The extract triggered the dysfunction of the mitochondria by damaging their membrane potential and inducing the apoptotic mechanism [53]

Chlorogenic acid is an interesting antioxidant molecule due to its wide spectrum of healthy effects on some pathologies, from diabetes to cardiovascular disease and cancer [54]. The oral administration of green coffee, particularly rich in chlorogenic acid, in hypertensive rats reduced the systolic blood pressure dose dependently without inducing any modification in the normotensive control group [55].

Food fortification is a valid method to solve micronutrients deficiencies. The most common example is the addition of iodine in cooking salt. In several countries, food fortification programs are becoming mandatory. In North and South America, the addition of folic acid to wheat flour is requested to reduce the risks of defects in the fetal neural structures [56,57]. Food fortification with iron produced a significant increase in hemoglobin and serum ferritin and reduced anemia and iron deficiency [58]. The fortified margarine with vitamin A showed its efficacy in increasing the levels of the vitamin in preschool children [59]. Sugar fortification with vitamin A is mandatory in Guatemala, where a tripling of the vitamin A is observed [56]. Polyphenol-fortified food showed their effects in fighting obesity by acting on the energy and food requests, lipid metabolism, and adipocyte differentiation [60]. These results indicate that food fortification is a good system to improve the diet quality.

## 5. Conclusions

The results of this work indicate that doughs enriched with PPP possess nutraceutical valuable compounds preserved during baking. The PPP bioactive compounds added in the biscuits were nontoxic and were able to counteract oxidative stress in Caco2 cells. Furthermore, the PPP compounds reduced the expression of proinflammatory genes in PBM cells. The doughs enriched with PPP have suitable mechanical properties that allow their lamination and provide them with adequate resistance during baking. All gathered evidence encourages further biological and clinical investigations to proceed.

## Figures and Tables

**Table 1:** Oligonucleotides sequences of forward and reverse primers used in PCR real time.

Items	Forward	Reverse
TNFα	5′-CCTCTCTCTAATCAGCCCTCTG-3′	5′-GAGGACCTGGGAGTAGATGAG-3′
L-1β	5′-CCTGTCCTGCGTGTTGAAAGA-3′	5′-GGGAACTGGGCAGACTCAAA-3′
L-8	5′-ATGACTTCCAAGCTGGCCGTGGCT-3′	5′-TCTCAGCCCTCTTCAAAAACTTCTC-3′
β-actin	5′-CATGTACGTTGCTATCCAGGC-3′	5′-CTCCTTAATGCTACGCACGAT-3′

**Table 2:** Total phenolic content in the PPP.

Molecules	Concentration (mg/kg)
Polyphenols *	16,220 ± 1206
Flavonoids	3300 ± 96
Antocyanins	363 ± 24

* Results are referred to the gallic acid calibration curve.

**Table 3:** Proposed compounds tentatively identified in pomegranate peel powder by HPLC-ESI-TOF-MS. The numbers correspond to the peaks illustrated in Figure 1.

Peak	RT	Meas. *m*/*z*	Calc. *m*/*z*	Err. [ppm]	Formula	Proposed Compound	Chemical Class
1	3.6	353.072	353.0725	1.6	C_12_H_17_O_12_	Sugar	Sugar
2	4.9	353.0716	353.0878	46	C_16_H_17_O_9_	Chlorogenic acid	Phenolic acids
3	5.5	191.0195	191.0197	0.9	C_6_H_7_O_7_	Citric acid	Organic acids
6	8.8	169.0127	169.0142	9.4	C_7_H_5_O_5_	Gallic acid	Phenolic acids
8	10.4	541.0266	541.026	−1	C_24_H_13_O_15_	Punicalagin α	Hydrolysable tannins
9	11.9	541.0277	541.026	−3.1	C_24_H_13_O_15_	Punicalagin β	Hydrolysable tannins
10	13	541.0263	541.026	−0.5	C_24_H_13_O_15_	Punicalagin γ	Hydrolysable tannins
11	15.4	633.0712	633.0733	3.4	C_27_H_21_O_18_	Corilagin	Hydrolysable tannins
12	15.6	463.0503	463.0518	3.3	C_20_H_15_O_13_	Ellagic acid hexoside	Hydrolysable tannins
15	19.5	447.0552	447.0569	3.9	C_20_H_15_O_12_	Ellagic acid-deoxyhexoside	Hydrolysable tannins
16	19.8	433.0387	433.0412	5.9	C_19_H_13_O_12_	Ellagic acid pentoside	Hydrolysable tannins
17	21.3	300.9973	300.999	5.6	C_14_H_5_O_8_	Ellagic acid	Hydrolysable tannins

**Table 4:** Total phenolic content in the heat treated PPP5 dough.

Molecules	Concentration (mg/kg)
Polyphenols	6087 ± 525
Flavonoids	294 ± 36
Antocyanins	137 ± 10

## Data Availability

All data are presented in the article.
